# Repair of α-particle-induced DNA damage in peripheral blood mononuclear cells after internal ex vivo irradiation with ^223^Ra

**DOI:** 10.1007/s00259-022-05860-3

**Published:** 2022-06-27

**Authors:** Lukas Göring, Sarah Schumann, Jessica Müller, Andreas K. Buck, Matthias Port, Michael Lassmann, Harry Scherthan, Uta Eberlein

**Affiliations:** 1 Department of Nuclear Medicine, University of Würzburg, Würzburg, Germany; 2 Bundeswehr Institute of Radiobiology affiliated to the University of Ulm, Munich, Germany

**Keywords:** DSB damage, Irradiation, α-Particle, γ-H2AX, Repair

## Abstract

**Purpose:**

As α-emitters for radiopharmaceutical therapies are administered systemically by intravenous injection, blood will be irradiated by α-particles that induce clustered DNA double-strand breaks (DSBs). Here, we investigated the induction and repair of DSB damage in peripheral blood mononuclear cells (PBMCs) as a function of the absorbed dose to the blood following internal ex vivo irradiation with [^223^Ra]RaCl_2_.

**Methods:**

Blood samples of ten volunteers were irradiated by adding [^223^Ra]RaCl_2_ solution with different activity concentrations resulting in absorbed doses to the blood of 3 mGy, 25 mGy, 50 mGy and 100 mGy. PBMCs were isolated, divided in three parts and either fixed directly (*d*-samples) or after 4 h or 24 h culture. After immunostaining, the induced γ-H2AX α-tracks were counted. The time-dependent decrease in α-track frequency was described with a model assuming a repair rate *R* and a fraction of non-repairable damage *Q*.

**Results:**

For 25 mGy, 50 mGy and 100 mGy, the numbers of α-tracks were significantly increased compared to baseline at all time points. Compared to the corresponding *d*-samples, the α-track frequency decreased significantly after 4 h and after 24 h. The repair rates R were (0.24 ± 0.05) h^−1^ for 25 mGy, (0.16 ± 0.04) h^−1^ for 50 mGy and (0.13 ± 0.02) h^−1^ for 100 mGy, suggesting faster repair at lower absorbed doses, while Q-values were similar.

**Conclusion:**

The results obtained suggest that induction and repair of the DSB damage depend on the absorbed dose to the blood. Repair rates were similar to what has been observed for irradiation with low linear energy transfer.

## Introduction

In nuclear medicine, the number of systemic treatments with α-particle emitters in patients with prostate cancer is increasing. The ALSYMPCA trial disclosed that [^223^Ra]RaCl_2_ treatment prolonged the lives of castration-resistant prostate cancer patients with widespread bone metastatic disease. Consequently, [^223^Ra]RaCl_2_ (Xofigo®, Bayer) obtained marketing authorization as the first α-particle emitting radiopharmaceutical [1, 2]. Furthermore, reports on compassionate use treatment with [^225^Ac]Ac-PSMA were published that describe the successful treatment in patients with prostate cancer metastases [3, 4] or in patients with neuroendocrine tumours [5].

α-Particles have a short range below 0.1 mm, a high linear energy transfer (LET) and have been found to induce complex DNA damage [6–8] and complex chromosome aberrations [9, 10]. When α-particle emitting radiopharmaceuticals such as [^223^Ra]RaCl_2_ are administered systemically by intravenous injection, target and non-target tissues will be exposed to ionising radiation [11]. After administration, the activity clears from the patients’ blood. Some activity remains in the blood for several days after the start of therapy, leading to prolonged internal irradiation with a decreasing absorbed dose rate [12, 13]. Consequently, the α-particles induce DNA double-strand breaks (DSBs) in the nuclei of the hit peripheral blood mononuclear cell (PBMC) [8, 14].

For the detection of DSBs in the low dose range < 100 mGy, the biomarkers γ-H2AX and 53BP1 are widely used [15]. Radiation-induced DSBs can be detected and quantified by microscopically visible DSB damage protein foci that display both γ-H2AX and 53BP1 [16–19]. DSB foci disappear by 53BP1 dissociation and γ-H2AX dephosphorylation after DSB repair has been completed [20, 21]. Low-LET electrons and photons induce localized focal assemblies relating to the generation of mostly simple isolated DSBs [22]. After irradiation with α-emitting radionuclides, not only distinct foci, but also γ-H2AX + 53BP1-containing DNA damage tracks, so-called α-tracks, are detected in hit cells’ nuclei [6, 23–25].

Ex vivo studies of ionising radiation-induced DSB formation in PBMCs indicate a linear relationship between the number of microscopically visible α-tracks and the absorbed dose to the blood after internal irradiation with α-emitters in the low dose range < 100 mGy [6, 7]. This observation was also confirmed in an in vivo study, when patient blood was examined in the first hours after [^223^Ra]RaCl_2_ administration [13]. However, the aforementioned studies mainly focused on the induction of DNA damage. The second study provided only limited data on the in vivo repair of the damage induced by the α-emitters in patients. Due to slow excretion of the activity from the blood in patients, the DNA damage induction competes with the DNA damage repair, thus affecting quantitative information on repair rates of DNA damage caused by α-particles. As high LET-induced complex DNA damage is considered difficult to repair [14, 26], a detailed analysis of these repair rates is of particular interest. Quantitative data on the repair after ex vivo internal irradiation with α-emitting radionuclides have to the best of our knowledge not been published so far.

Therefore, the aim of this study was to analyse ex vivo the induction and repair of DSBs in isolated PBMCs after an internal ex vivo irradiation of whole blood with [^223^Ra]RaCl_2_ with nominal absorbed doses between 3 mGy and 100 mGy.

## Materials and methods

### Blood sampling, irradiation and isolation of PBMCs

Ten healthy volunteers (5 female and 5 male) aged between 23 and 64 years were included in the study. On the day of the test, 35 ml of blood were taken from each subject using Li-Heparin blood-collecting tubes (S-Monovette®; Sarstedt). The blood was then divided into five samples of 7 ml each. One of the five samples remained unirradiated and thus served as a control. Each of the other samples was supplemented with 1 ml [^223^Ra]RaCl_2_ solution diluted in phosphate-buffered saline (PBS) in different concentrations, resulting in four different predetermined absorbed doses to the blood: 3 mGy, 25 mGy, 50 mGy and 100 mGy. During incubation at 37 °C for 1 h, the samples were on a roller mixer to ensure uniform irradiation from ^223^Ra and its progeny. Subsequently, 1 ml of each sample was pipetted into round-bottom tubes to determine the exact activity concentration, while the remaining sample was transferred to CPT Vacutainer tubes (BD) and centrifuged according to the manufacturer’s instructions in order to separate the PBMCs from the rest of the blood and the radioactive solution. The isolated PBMCs of each of the five blood samples were split into three parts and washed twice in PBS, so that less than 0.1% of the initially added activity was still detectable in the cell pellet. Then, PBMCs were fixed either directly by adding 70% ice-cold ethanol solution (*d* = 0 h) to investigate DNA damage induction (d-sample) or after 4 h or 24 h of culture in RPMI medium containing HEPES (Life Technologies) to determine DNA repair. Thus, a total of 15 cell samples per volunteer were processed. The cells were stored at − 20 °C before they were shipped to the Bundeswehr Institute of Radiobiology in Munich, Germany, for further processing.

### Immunofluorescent staining and evaluation of DNA damage

The immunofluorescent staining of the ethanol-fixed PBMCs with γ-H2AX and 53BP1 antibodies was carried out as described by Scherthan et al. [27]. The classification and evaluation of DNA damage followed the protocol described by Schumann et al. [6]. γ-H2AX-positive tracks were considered as “α-tracks” and were counted manually in 500 cells per sample by the same experienced investigator (H.S.). From this, the average number of α-tracks per 100 cells and the corresponding counting errors (by assuming a Poisson distribution) were calculated.

### Activity quantification and absorbed dose calculation

An aliquot of 1 ml of each irradiated blood sample was measured in a calibrated, high-purity germanium detector (Canberra). The activity in each blood sample was determined by evaluating three different γ-emission lines of ^223^Ra and its progeny as described by Schumann et al. [6]. The activity concentration was then decay corrected to the start of incubation.

For the calculation of the absorbed dose coefficient, it was assumed that all α-particles emitted by ^223^Ra and its progeny, i.e. four α-particles per decay, deposit their energy locally in the blood sample. The number of ^223^Ra disintegrations within 1 h in 1 ml of blood was calculated. This resulted in an absorbed dose coefficient of 15.5 mGy kBq^−1^, as described by Schumann et al. [6]. To calculate the absorbed dose to the blood, this absorbed dose coefficient was multiplied by the activity concentration in the corresponding blood sample.

### Modelling of DNA damage repair

Schumann et al. [28] described the reduction of radiation-induced γ-H2AX + 53BP1 foci after internal irradiation with ^131^I in analogy to Lobachevsky et al. [29]. This approach can also be used to model the number of α-tracks per 100 cells $N(t)$ at time $t$:

1 $$N\left(t\right)={N}_{0}\cdot \left(\left(1-Q\right)\cdot\mathrm{exp}\left(-Rt\right) +Q\right)$$

Here, ${N}_{0}$ is the maximum number of α-tracks per 100 cells and $Q$ the fraction of unrepaired α-tracks, i.e. the residual damage. $R$ denotes the repair rate in h^−1^.

### Statistical analysis

OriginPro 2019b (Origin Lab Corporation) was used for statistical analysis and plotting. The Shapiro-Wilk test was used to test the data for normal distribution. To compare datasets, paired-sample *t*-tests (for normally distributed data) and Wilcoxon signed-rank tests (for not normally distributed data) were conducted. The results were considered as statistically significant for *p* < 0.05.

## Results

### Absorbed dose-dependent α-track induction and decrease

**Fig. 1:**
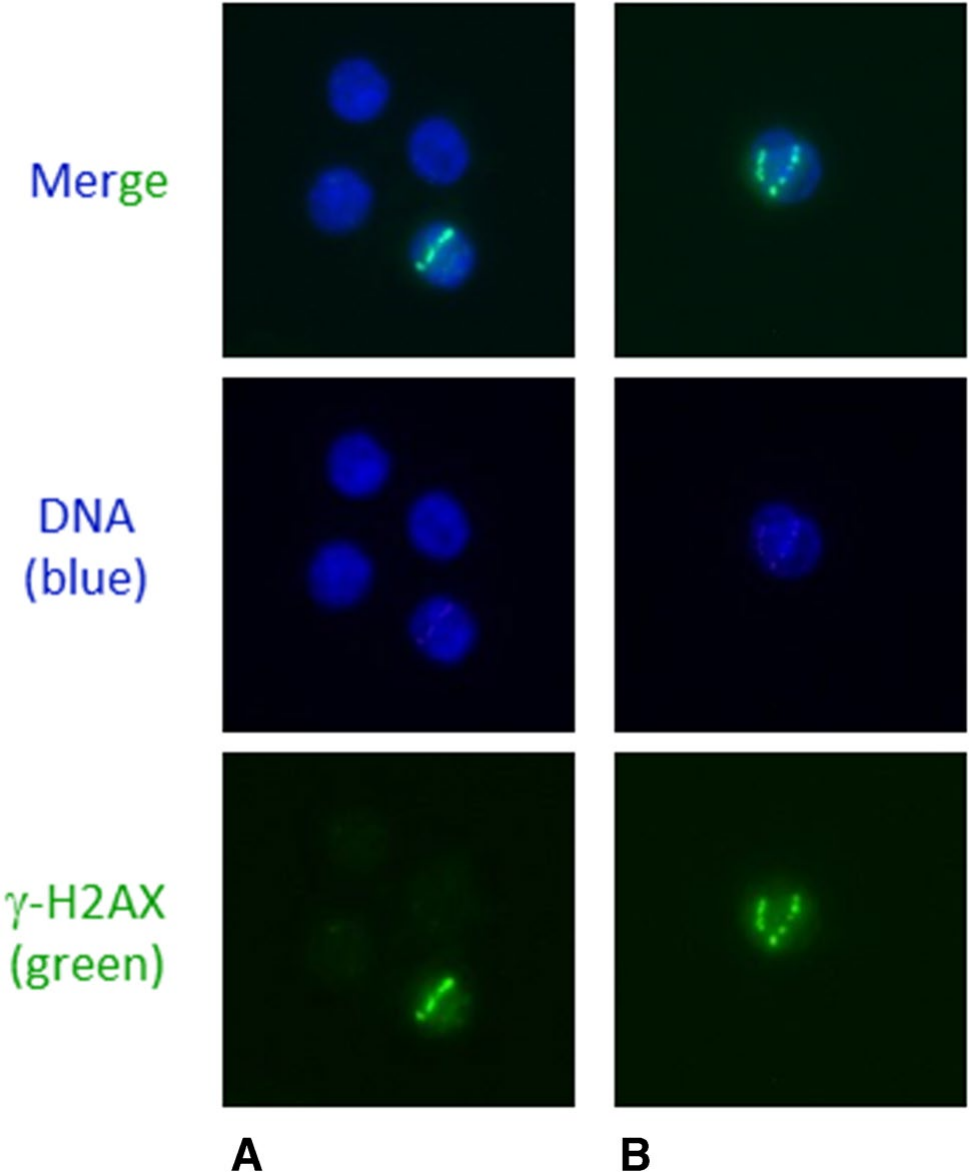
Selection of PBMC nuclei with α-particle hits (α-tracks) after irradiation with [^223^Ra]RaCl_2_. DNA was stained with DAPI (blue) and γ-H2AX as DNA damage marker (green). All images show the linear morphology of the damage clusters. Additionally, γ-H2AX signals across the entire affected nuclei can be seen. This is a typical finding after exposure of cells to high LET irradiation [13, 42]. In all unaffected nuclei, almost no γ-H2AX is visible. **A** One of the four cell nuclei contains a single α-track. **B** Rare event of two α-tracks within one nucleus

**Fig. 2:**
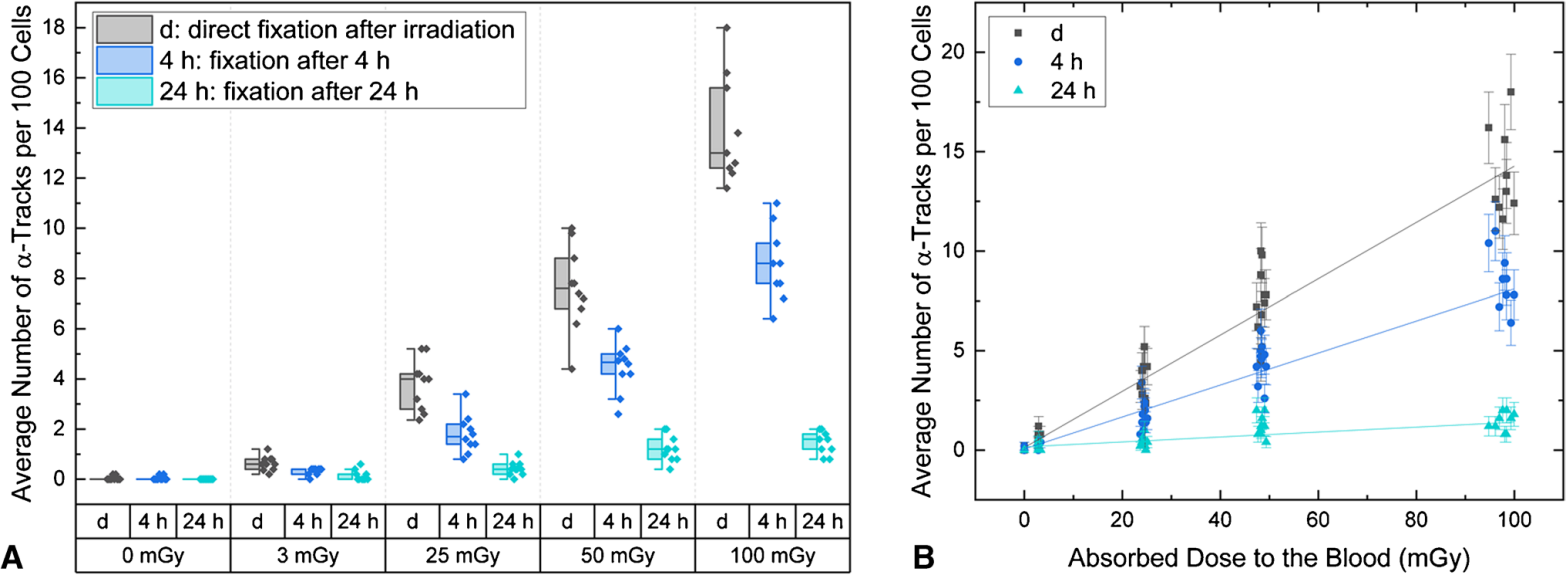
Average number of α-tracks per 100 cells for each absorbed dose value and for each of the three time points (*d*, 4 h and 24 h) studied (*n* = 10). For each data point, α-tracks were counted in 500 cells. **A** Boxplot of all data points. **B** Plot of the absorbed dose-dependency with separate linear fits for the three time points

The calculated values of the absorbed dose to the blood of the ten experiments conducted are given in Table 1. Examples of nuclei with α-tracks are shown in Fig. 1. The average number of α-tracks in 100 cells for each absorbed dose value and for each of the three time points (d, 4 h, and 24 h) studied is shown in the boxplot in Fig. 2A, and the corresponding values are given in Table 1. In 26 of the 30 unirradiated samples, no α-tracks were detected, whereas in the other four samples, one α-track per 500 cells was observed. This corresponds to 0.03% α-tracks in the baseline samples. A significant increase of the average number of α-tracks was observed for all irradiated *d*-samples compared to baseline. For the absorbed doses to the blood of 25 mGy, 50 mGy and 100 mGy, the average number of α-tracks decreased significantly after 4 h and after 24 h cell culture in RPMI medium. After 4 h, 48% to 62% of the initial numbers of α-tracks in the *d*-samples were still detectable. After 24 h, this number decreased to 10% to 16% (Table 2). For 3 mGy, a similar trend of α-track reduction was visible (4 h: 50% persistent α-tracks; 24 h: 22% persistent α-tracks), however, not being significant due to the high uncertainty of the comparatively low α-track numbers. The number of α-tracks after 24 h in culture was still significantly increased compared to baseline in the 25 mGy, 50 mGy and 100 mGy samples, indicating that DNA repair in some cells with α-tracks is not completed after 24 h.

**Table 1:** Absorbed dose to the blood, activity concentration and average number of α-tracks per 100 cells. In each case, the median value (including the minimum and maximum value) is given and below, if applicable, the mean value with the standard deviation

Nominal absorbed dose to the blood (mGy)	Nominal activity concentration in the blood (kBq ml^−1^)	Absorbed dose to the blood (mGy)	Average number of α-tracks in 100 cells		
			*d*	4 h	24 h
0 (baseline)	0	0	0.0 (0.0–0.2)	0.0 (0.0–0.2)	0.0 (0.0–0.0)
3	0.2	2.9 (2.8–3.3)	0.6 (0.2–1.2)	0.4 (0.0–0.8)	0.0 (0.0–0.6)
25	1.6	24.4 (23.7–25.1) 24.4 ± 0.4	4.0 (2.4–5.2) 3.8 ± 1.0	1.7 (0.8–3.4) 1.9 ± 0.8	0.4 (0.0–1.0) 0.5 ± 0.3
50	3.2	48.4 (47.4–49.4) 48.4 ± 0.6	7.3 (4.4–10.0) 7.3 ± 1.8	4.7 (2.6–5.2) 4.4 ± 0.8	1.2 (0.4–2.0) 1.2 ± 0.5
100	6.5	97.8 (94.8–99.9) 97.7 ± 1.6	13.0 (11.6–18.0) 13.9 ± 2.2	8.6 (6.4–11.0) 8.6 ± 1.5	1.6 (0.8–2.0) 1.4 ± 0.5

**Table 2:** Fit parameters of the DNA damage repair model and percentage of persistent α-tracks as a function of the repair time

Nominal absorbed dose to the blood (mGy)	Persistent α-tracks (%)*		Fit parameters			
	4 h	24 h	*N*_0_	*R* (h^−1^)	*Q*	*r*^2^
25	48	11	3.51 ± 0.29	0.24 ± 0.05	0.09 ± 0.03	0.83
50	58	16	7.24 ± 0.48	0.16 ± 0.04	0.12 ± 0.03	0.88
100	62	10	13.66 ± 0.61	0.13 ± 0.02	0.06 ± 0.03	0.96

^*^Relative to the initial number of α-tracks in the directly fixed samples (*d*)

The absorbed dose-dependent α-track frequency is plotted in Fig. 2B. Linear fits to the pooled data for the three time points investigated demonstrated that the α-track induction is linearly dependent on the absorbed dose to the blood in the directly fixed samples (*r*^2^ = 0.95) and in the samples fixed after 4 h (*r*^2^ = 0.91) (Table 3). After 24 h, linearity between the absorbed dose to the blood and the number of residual α-tracks is no longer evident (*r*^2^ = 0.56). The coefficients of determinations, the slope and the intercept values of the linear fits are listed in Table 2.

**Table 3:** Results of the linear fits for the three time points (*d*, 4 h and 24 h) investigated

	Slope (mGy^−1^)	Intercept	r^2^
*d*	0.141 ± 0.005	0.131 ± 0.084	0.95
4 h	0.080 ± 0.004	0.062 ± 0.073	0.91
24 h	0.012 ± 0.002	0.162 ± 0.067	0.56

### Modelling of DNA damage repair

**Fig. 3:**
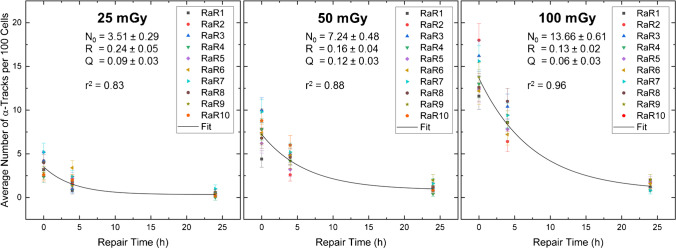
Average number of α-tracks per 100 cells as a function of the repair time for 25 mGy, 50 mGy and 100 mGy. The black curves represent population-based fits according to Eq. 1. The resulting fit parameters, i.e. the maximum number of α-tracks *N*_0_, the repair rate R in h^−1^ and the fraction of unrepaired α-tracks Q, are given in each case. Additionally, the coefficient of determination *r*^2^ is given

Figure 3 shows the time-dependent α-track reduction for the different absorbed doses to the blood (25 mGy, 50 mGy and 100 mGy) including monoexponential fits to the pooled data of all volunteers according to Eq. 1. Data on the 3 mGy samples were excluded from these analyses due to low statistical power. The α-track repair rates $R$ were (0.24 ± 0.05) h^−1^ for 25 mGy, (0.16 ± 0.04) h^−1^ for 50 mGy and (0.13 ± 0.02) h^−1^ for 100 mGy and thus decrease with increasing absorbed doses, suggesting more rapid repair at lower absorbed doses. The fractions of unrepaired α-tracks $Q$ were 0.09 ± 0.03 for 25 mGy, 0.12 ± 0.03 for 50 mGy and 0.06 ± 0.03 for 100 mGy and therefore similar for all absorbed dose values within the range of the errors. All fit parameters are listed in Table 2.

## Discussion

In this study, the time course of α-particle-induced DNA damage was systematically investigated by internal ex vivo irradiation of blood samples. This setup was chosen to mimic the irradiation in vivo in a patient during radionuclide therapy with an α-emitter, such as the radiopharmaceutical [^223^Ra]RaCl_2_ used here. The absorbed dose range between 3 mGy and 100 mGy was chosen since it reflects the absorbed doses to the blood that can be expected during corresponding radionuclide therapies [13, 30, 31]. For this dose range, there were no data yet that systematically describe the repair of DNA DSBs after internal irradiation with α-emitters.

The duration of the internal irradiation process in this study was set at 1 h in order to be able to examine several blood samples with different absorbed doses at the same day. Longer irradiation durations might have better mimicked the behaviour in the patient but would have resulted in a more complicated experimental setup, which in turn would have meant that fewer samples could have been processed.

After 1 h irradiation, the average number of α-tracks per 100 cells increased linearly with the absorbed dose to the blood and was (0.141 ± 0.005) mGy^−1^. This value is in the same order of magnitude, but lower than the expected value of (0.222 ± 0.014) mGy^−1^ according to an ex vivo calibration curve [6]. The reason for the different induction rates could be the slightly different protocol of the blood sample preparation as well as the enumeration of the α-tracks. While Schumann et al. evaluated 100 cells per sample [6], 500 cells per sample were evaluated in the present study, thereby reducing counting errors. Moreover, the absorbed dose ranges in the two studies were slightly different. The induction of α-tracks was also investigated in vivo by analysing the α-track frequency in PBMCs of patients during therapy with [^223^Ra]RaCl_2_ [13]. In the in vivo study, significantly increased α-track frequencies were observed 1.5 h after administration of [^223^Ra]RaCl_2_, at absorbed doses to the blood of only 0.5 mGy to 3.2 mGy [13]. The number of α-tracks per 100 cells induced after 1.5 h in vivo (mean: 0.93 ± 0.33) is similar to the number of α-tracks in the directly fixed 3 mGy samples that ranged between 0.2 and 1.2 [13].

After 4 h cell culture, a significant reduction of the α-track frequency compared to the directly fixed PBMCs was demonstrable for absorbed doses ≥ 25 mGy. This decline in the number of α-tracks can be interpreted as the progression of DNA repair. Alternatively, cells with α-particle damage might have been removed by cell death. However, we only occasionally noted PBMCs displaying pan-γ-H2AX pattern (< 0.7%) indicative of cell death [32, 33] in our samples without significant differences among the time points and absorbed doses investigated. Therefore, it is unlikely that an elimination of damaged cells influenced the reduction of the frequency of α-tracks over time.

After 24 h cell culture, up to 89% of the α-particle-induced damage was repaired: Only 11% to 23% of the initially detected α-tracks remained. The fraction of remaining α-tracks after 24 h matches the assumption that approximately 15% of radiation-induced DSBs are repaired with slow kinetics and require ATM and the nuclease Artemis [34, 35]. The decline in the α-track frequency ex vivo contrasts the observations in vivo [13]. In the in vivo study, the average number of α-tracks per 100 cells 24 h after the administration of [^223^Ra]RaCl_2_ (at absorbed doses to the blood below 11 mGy) ranged between 0.82 and 2.37 and is similar to the α-track numbers within in the first hours after therapy start [13]. In comparison to the situation ex vivo, the irradiation in vivo is not stopped after 1 h, but continues. The absorbed dose increases further in vivo, albeit at a decreasing dose rate. This may explain why the reduction of the α-track frequency observed in this ex vivo study was not seen in vivo.

The decrease of the number of α-tracks with time can be adequately described by a model with a monoexponential decay function. However, the number of time points examined in this study was restricted to 3 due to the limited amount of blood we could draw from each volunteer. This limitation also meant that separate fits could not be performed for the individual subjects, allowing only a general fit of the pooled data.

From the fit parameters, it is evident that a certain proportion Q of DNA damage, ranging from 5% to 11% in the studied dose range, remained unrepaired. This matches the observation that 11% to 23% of the α-tracks are still detectable after 24 h repair time ex vivo, and it also agrees with the observations of incomplete repair of the complex DNA damage even days and weeks after therapy start in vivo [13].

The model presented here was also used to describe the reduction of radiation-induced γ-H2AX + 53BP1 DSB foci after internal ex vivo irradiation with the β^−^-emitter ^131^I [28]. For an absorbed dose to the blood of 50 mGy, the foci repair rate was *R* = (0.28 ± 0.03) h^−1^ compared to (0.16 ± 0.04) h^−1^ for α-tracks in the present study [28]. This indicates that the repair of α-particle-induced complex DNA damage progresses slower than the repair of β-emitter-induced simple DSB damage. The fraction of unrepaired radiation-induced γ-H2AX + 53BP1 foci after irradiation with ^131^I was *Q* = 0.06 ± 0.02 [28]. This value is similar to the *Q*-values of the remaining α-tracks obtained in the present study, suggesting that the percentage of residual damage does not differ for ex vivo irradiation with α- or β-emitting radionuclides in the absorbed dose range studied. The latter may be puzzling, but given that DSB repair is difficult or retarded in the heterochromatin compartment of the nucleus [34–37], it may well be that the cells displaying persistent DNA damages may be those that carry DSBs buried in heterochromatin [35]. It should also be noted that the parameter *Q* describes the relative proportion of residual damage and does not represent an absolute value. Since the number of DSBs per track can be estimated to be 12 ± 9 [23], the absolute number of persistent damages after α-irradiation is higher than after low-LET irradiation. This agrees with observations of persistent complex DNA damage after high-LET irradiation [8, 14, 38]. The fate of the α-particle-induced persistent damage in PBMCs at time points > 24 h remains to be determined.

The observation that the α-track repair rates differ for the various absorbed doses investigated indicates that DSB repair depends on the absorbed dose. The decrease of the *R*-values with increasing absorbed doses above 25 mGy suggests more rapid repair at lower absorbed doses after internal irradiation. This is in contrast to studies investigating DSB repair after external low-LET irradiation which showed a compromised DSB repair rate at very low doses [39–41]. Rothkamm and Löbrich reported a decreasing repair capacity for X-ray doses below 20 mGy and a complete lack of repair at 1.2 mGy [39]. In the current study, we did not obtain significant results when investigating the repair of DSB damage induced by internal irradiation with 3 mGy absorbed dose to the blood. A potential explanation is that the uncertainty in the α-track frequency was too high in 500 evaluated cell nuclei. The study of the repair after α-particle exposure in the absorbed dose range below 25 mGy, therefore, requires further studies with a significantly higher number of evaluated cells. However, this will only be possible upon the development of automatic counting systems that can reliably quantify α-tracks in larger cell numbers per sample.

## Conclusion

The time- and dose-dependency of track-like DSB damage induced by internal irradiation with the clinically used α-emitter [^223^Ra]RaCl_2_ was investigated ex vivo under defined conditions and modelled using appropriate fit functions. The obtained results suggest that induction and repair of the DSB damage depend on the absorbed dose to the blood, yielding repair rates similar to what has been observed for low-LET irradiation.
